# Multimodal omics analysis of the EGFR signaling pathway in non-small cell lung cancer and emerging therapeutic strategies

**DOI:** 10.32604/or.2025.059311

**Published:** 2025-05-29

**Authors:** YUZHENG LI, LILI YU, SHIYAO ZHOU, HUA ZHOU, QIBIAO WU

**Affiliations:** 1 Faculty of Chinese Medicine, State Key Laboratory of Quality Research in Chinese Medicine, and University Hospital, Macau University of Science and Technology, Macao, 999078, China; 2 Chinese Medicine Guangdong Laboratory (Hengqin Laboratory), Guangdong-Macao In-Depth Cooperation Zone in Hengqin, Zhuhai, 519000, China; 3 State Key Laboratory of Traditional Chinese Medicine Syndrome, Guangdong Provincial Hospital of Chinese Medicine, Guangdong Provincial Academy of Chinese Medical Sciences, The Second Affiliated Hospital of Guangzhou University of Chinese Medicine, Guangzhou, 510006, China

**Keywords:** Cancer therapy, Epidermal growth factor receptor (EGFR), Multiomics technologies, Non-small cell lung cancer (NSCLC), Targeted therapies

## Abstract

**Background:**

Non-small cell lung cancer (NSCLC) involves complex alterations in the epidermal growth factor receptor (EGFR) signaling pathway. This study aims to integrate multimodal omics analyses to evaluate and enhance EGFR-targeted therapies.

**Methods:**

We reviewed and synthesized omics data—including genomics, transcriptomics, proteomics, epigenomics, and metabolomics data—related to the EGFR pathway in NSCLC, examined the clinical outcomes of current therapies and proposed new treatment strategies.

**Results:**

Integrated omics analyses revealed the multifaceted role of EGFR in NSCLC. Transcriptomic analysis revealed gene expression alterations due to EGFR mutations, with upregulation of oncogenes and downregulation of tumor suppressors. Proteomics revealed complex interactions within the EGFR network, revealing cross-talk with other receptors. Epigenomics highlighted the impact of DNA methylation and histone modifications on EGFR and its downstream genes, whereas metabolomics demonstrated shifts in metabolic patterns essential for tumor growth.

**Conclusion:**

This study highlights the critical role of multimodal omics in understanding the molecular landscape of NSCLC, offering insights into more effective, personalized therapies. Future advancements in omic technologies and analysis are expected to significantly enhance NSCLC diagnosis and treatment.

## Introduction

Non-small cell lung cancer (NSCLC) accounts for approximately 85% of all lung cancer diagnoses [1]. Despite advancements in traditional therapies, the prognosis for patients with advanced NSCLC remains poor, primarily due to the heterogeneity of the tumors [2]. Targeted therapies against epidermal growth factor receptor (EGFR) have substantially improved outcomes for patients harboring EGFR mutations [3]. Significant progress in this field was demonstrated by the FLAURA2 trial in 2023, which confirmed the efficacy of osimertinib, a third-generation EGFR tyrosine kinase inhibitor (TKI), in combination with chemotherapy, achieving a progression-free survival (PFS) of 25.5 months in patients with advanced EGFR-mutant NSCLC [4]. In early 2024, findings from the MARIPOSA study further supported the effectiveness and safety of combining amivantamab with lazertinib in this patient cohort [5].

Recent trials have investigated the synergistic effects of combining osimertinib with Mesenchymal-Epithelial Transition (MET) inhibitors to overcome MET amplification-mediated resistance, a common mechanism in EGFR-TKI-resistant NSCLC. The TATTON trial and the ongoing SAVANNAH trial have shown promising results for osimertinib combined with savolitinib, a selective MET inhibitor, in patients with MET-driven resistance. Interim data from the SAVANNAH trial in 2024 demonstrated a clinically meaningful response rate in patients with acquired MET amplification, suggesting this combination as a potential strategy for overcoming MET-related resistance [6,7]. Other trials, such as the ORCHARD trial, are evaluating osimertinib-based combination therapies tailored to the molecular profile of resistance in NSCLC patients [8].

However, the benefits of targeted therapies are not universal, with some patients developing acquired resistance [9]. To address this challenge, we employ a comprehensive array of advanced bioinformatics tools and machine learning algorithms to integrate and analyze multimodal omics data, including genomics, transcriptomics, proteomics, epigenomics, and metabolomics. By applying these sophisticated technologies, we systematically identify key molecular drivers and resistance mechanisms in NSCLC, thereby guiding the development of targeted therapeutic strategies [10]. Genomic data are processed using the Genome Analysis Toolkit (GATK) for variant calling and ANNOtate VARiation (ANNOVAR) for the annotation of genetic variants critical to NSCLC [11,12]. Transcriptomic data are mapped with the Spliced Transcripts Alignment to a Reference (STAR) aligner and quantified using Cufflinks to identify gene expression alterations linked to EGFR mutations [13,14]. Proteomic analyses are performed using MaxQuant for peptide identification and quantification, integrated with Perseus for statistical analysis and data visualization, aiding in the discovery of protein expression patterns and potential biomarkers [15]. Epigenomic data are analyzed using Bismark, enhancing our understanding of the epigenetic landscape shaped by EGFR activity [16]. Metabolomic profiling, conducted with MetaboAnalyst alongside Liquid Chromatography-Mass Spectrometry (LC-MS) and Gas Chromatography-Mass Spectrometry (GC-MS), identifies shifts in metabolite profiles indicative of the biochemical activities within NSCLC cells [17]. Furthermore, data integration is facilitated by the Galaxy platform, which provides a comprehensive framework for data management and visualization [18]. Predictive models of disease progression and response to therapy are constructed using Python-based machine learning algorithms, leveraging libraries such as scikit-learn [19].

This review aims to meticulously summarize the outcomes of multimodal omics analyses of the EGFR signaling pathway in NSCLC, assess the efficacy and limitations of existing EGFR-targeted therapies, and discuss innovative therapeutic strategies based on omics data. These include the development of new-generation EGFR inhibitors and combination treatment plans, with a view towards personalized therapy. Through this comprehensive analysis, we endeavor to illuminate the molecular mechanisms underlying NSCLC and contribute to the advancement of more effective treatment modalities.

### The role of the EGFR signaling pathway in non-small cell lung cancer

EGFR mutations play crucial roles in NSCLC, particularly in Asian populations. The spectrum of EGFR mutations primarily includes deletions in exon 19, L858R point mutations in exon 21, insertions in exon 20, mutations in exon 18, and some rare mutations. The overall prevalence of EGFR mutations in Asian populations is approximately 30%–50%, compared to 10%–20% in Western populations. Deletions in exon 19 and L858R mutations are the most prevalent, accounting for 80%–90% of cases in Asians. Younger individuals, particularly women under 40 years in Asia, exhibit a higher mutation rate. Environmental factors such as air quality and exposure to carcinogens also significantly influence mutation prevalence, underscoring the need for targeted prevention strategies [20].

EGFR mutation prevalence exhibits significant geographic and ethnic variability [21,22]. Notably, East Asian countries such as China, Japan, and South Korea report higher mutation rates than those observed in the West. Research indicates that genetic predispositions, environmental exposures, and lifestyle choices all contribute to these disparities. Notably, there are also variations within Asia: Southeast Asian countries have slightly lower EGFR mutation rates than East Asian countries do, whereas South Asian countries fall between East Asian countries and West Asian countries. Recent studies have also revealed that while the EGFR mutation rates in Africa and Latin America are lower than those in Asia, they are still higher than those in Europe and North America.

Demographic characteristics also play important roles in the distribution of patients with EGFR mutations [23]. Women, nonsmokers, and patients with adenocarcinoma present higher frequencies of EGFR mutations, a trend present in all geographic regions but more pronounced in Asia. Age factors are also significant, with recent research indicating that younger patients, especially those under 40 years, may have higher rates of EGFR mutations, particularly young Asian women. In multiethnic countries such as the USA, Asian Americans have a noticeably higher frequency of EGFR mutations than other ethnicities do, further emphasizing the importance of genetic factors in the prevalence of EGFR mutations.

**Figure 1:**
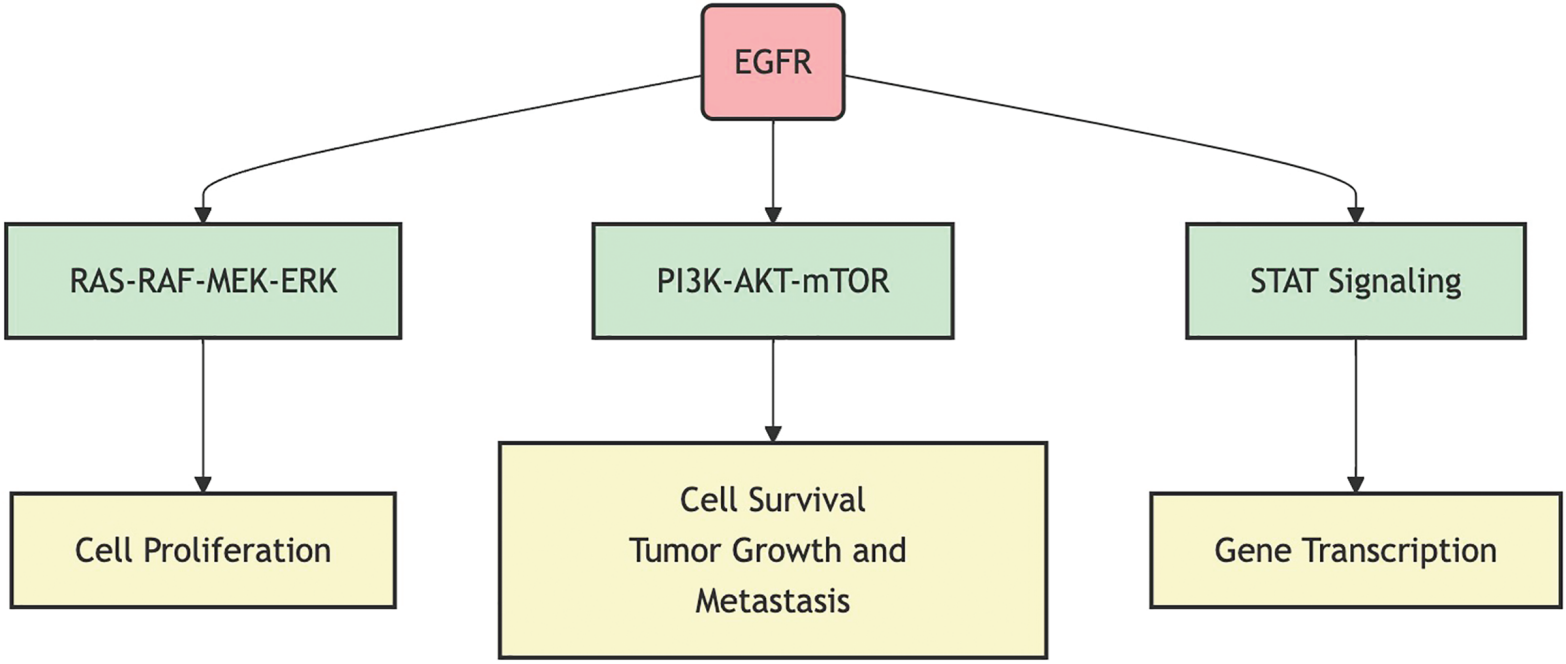
EGFR signaling pathway and its primary downstream effects.

Aberrant activation of the EGFR signaling pathway leads to alterations in multiple downstream signaling pathways, including the RAS-RAF-MEK-ERK pathway, the PI3K-AKT-mTOR pathway, the STAT signaling pathway, and the PLCγ-PKC pathway [24]. The abnormal activation of these downstream pathways not only promotes tumor cell proliferation and survival but is also closely associated with tumor invasion, metastasis, and angiogenesis. Fig. 1 illustrates the primary downstream pathways activated by EGFR signaling and their biological effects, including cell proliferation, survival, and gene transcription. Additionally, aberrant activation of the EGFR signaling pathway can affect the tumor microenvironment, such as by promoting the secretion of angiogenic factors, thereby increasing the blood supply to the tumor [25], or by modulating the expression of immune-suppressive factors, helping tumor cells evade immune surveillance [26]. A deep understanding of the complex role of the EGFR signaling pathway in NSCLC is crucial for developing new treatment strategies and overcoming drug resistance.

## Comprehensive Multimodal Omics Profiling of the EGFR Signaling Pathway

### Characterization of EGFR mutation spectrum and prevalence

The overall frequency of EGFR mutations in NSCLC ranges from 10%–35%, with significant variability across different populations. The most common activating mutations of EGFR are exon 19 deletions (approximately 45%) and exon 21 L858R point mutations (approximately 40%), both of which respond well to EGFR-TKI treatment [22]. Other important but less common mutations include exon 18 G719X mutations (approximately 3%), exon 20 insertions (4%–10%, typically resistant to first- and second-generation EGFR-TKIs), and T790M mutations (primary <5%, acquired resistance up to 60%) [27]. Table 1 summarizes the distribution of key EGFR mutation types, their frequencies, and geographic characteristics.

**Table 1:** Distribution of EGFR mutations

EGFR mutation type	Frequency	Geographic distribution characteristics
Exon 19 deletion	45%	Asian populations: 30%–50%
Exon 21 L858R point mutation	40%	Western populations: 10%–20%
Exon 18 G719X mutation	3%	Less common, but may be more prevalent in certain populations
Exon 20 insertion	4%–10%	
T790M mutation	Primary <5%	
	Acquired resistance up to 60%	

Recent advances in circulating tumor DNA (ctDNA) analysis have provided a noninvasive method for dynamically monitoring the EGFR mutation status, especially the emergence of resistance-related mutations. Studies on the EGFR mutation spectrum and frequency are crucial for understanding the molecular pathology of NSCLC, guiding the selection of targeted therapies, and developing new drugs. With technological progress, our understanding of the EGFR mutation spectrum continues to deepen, pushing the development of precision treatment for NSCLC.

### EGFR-associated gene expression patterns

EGFR mutation or activation extensively affects whole-genome expression in NSCLC. Through large-scale transcriptomic analyses, researchers have identified a series of gene expression patterns associated with the EGFR signaling pathway. The activation of EGFR leads to the upregulated expression of genes that promote cell proliferation and survival, such as cell cycle-related genes (CCND1 and CDK4), antiapoptotic genes (BCL2 and BIRC5), and angiogenesis-related genes (VEGFA and ANGPT2) [28]. Moreover, genes that inhibit tumor growth, including those that promote apoptosis (BAX and CASP3) and are related to cell adhesion (CDH1 and ITGB1), are downregulated [29].

Activation of the EGFR signaling pathway also triggers feedback regulatory mechanisms, such as the upregulation of the negative feedback regulators DUSP6 and SPRY2 [30]. Moreover, different EGFR mutation types may lead to slightly varied gene expression profiles, potentially explaining their differences in clinical manifestations and treatment responses. To combat the complexity of resistance mechanisms in NSCLC, our therapeutic approach includes a multi-target strategy. This involves targeting not only EGFR but also other critical nodes within the signaling network, such as MET, Human Epidermal Growth Factor Receptor 2(HER2), and AXL Receptor Tyrosine Kinase (AXL), simultaneously. By inhibiting multiple pathways, we aim to prevent the cancer cells’ ability to compensate for one blocked pathway by upregulating another. This strategy is supported by our omics analysis, which maps the interactions and redundancies within the cellular signaling networks, providing a blueprint for targeted multi-pronged treatment approaches [31]. In response to EGFR-TKI therapy, certain tumor cells may exhibit upregulation of genes associated with alternative signaling pathways that bypass EGFR blockade. Notably, MET, HER2, and AXL are critical in mediating these resistance mechanisms. These genes encode receptor tyrosine kinases that can activate parallel pathways, sustaining cell survival and proliferation despite EGFR inhibition. Detailed discussions on the interaction dynamics of these proteins and their contributions to therapeutic resistance are crucial for understanding resistance patterns and devising effective combination therapies [32]. Activation of the EGFR pathway also affects the expression and activity of various transcription factors, such as increasing c-MYC and E2F1 activity, while p53 activity may be inhibited [33]. In recent years, studies have revealed that noncoding RNAs play a significant role in the EGFR signaling pathway. Certain microRNAs may directly regulate the expression of EGFR, while some long noncoding RNAs may be involved in regulating the expression of genes downstream of EGFR [34].

### EGFR pathway protein interaction network

The EGFR pathway protein interaction network is key to understanding the mechanisms of EGFR signal transduction. Through proteomics and interactomics studies, scientists have constructed a complex and detailed map of the EGFR signaling network, revealing how EGFR regulates various biological processes through interactions with multiple proteins.

The binding of EGFR to its ligands, such as EGF, TGFα, and HB-EGF, is the starting point of the entire signaling network, leading to receptor dimerization and autophosphorylation, thus activating downstream signaling pathways. Activated EGFR interacts with adaptor proteins (such as GRB2 and SHC1) and signal transduction molecules, recruiting downstream effector molecules. Important downstream effector molecules include PIK3CA and STAT3, which activate the PI3K-AKT-mTOR pathway and the STAT signaling pathway, respectively [35].

EGFR also undergoes cross-activation with other receptor tyrosine kinases (such as HER2, MET, and IGF1R), forming a complex signaling network. At the cellular membrane level, EGFR interacts with cell adhesion and cytoskeletal proteins, affecting cell–cell and cell-matrix adhesion as well as cell migration [36]. EGFR mutations may alter the interaction patterns with certain proteins, potentially explaining the carcinogenic mechanisms and resistance of some mutations. During the acquisition of drug resistance, significant changes in the EGFR signaling network, such as new interactions caused by MET amplification or AXL activation, occur [37].

Recent studies have focused on the dynamics of EGFR signaling complex formation and dissociation and how its spatial distribution within cells affects signal strength and specificity [38]. Moreover, the EGFR pathway is regulated by multiple negative feedback mechanisms, such as receptor degradation mediated by the E3 ubiquitin ligase CBL and dephosphorylation of EGFR by the phosphatase PTPN1 [39].

### EGFR-related epigenetic modification features

EGFR-related epigenetic modifications are crucial for understanding the regulation of the EGFR gene and the mechanisms of tumor development and progression. These modifications include DNA methylation, histone modifications, chromatin remodeling, and noncoding RNA regulation, all of which play key roles in the regulation of EGFR gene expression, the activation of signaling pathways, and the development of resistance.

The DNA methylation status of the EGFR gene promoter region is closely related to its expression level. Low levels of methylation are generally associated with increased expression of EGFR, possibly due to decreased expression or activity of DNA methyltransferases [40]. Histone modifications also play a significant role in regulating EGFR gene expression; active marks such as H3K4me3 and H3K27ac are generally associated with high gene expression, whereas repressive marks such as H3K27me3 may lead to the suppression of expression [41].

Chromatin remodeling complexes such as SWI/SNF and NuRD play roles in regulating the accessibility of the EGFR gene [42]. Noncoding RNAs, particularly miRNAs and lncRNAs, are also involved in the epigenetic regulation of EGFR. Certain EGFR mutations may alter related epigenetic modification patterns, affecting gene expression or protein activity. Dynamic changes in epigenetic modifications during EGFR-TKI treatment may influence the development of resistance. Additionally, the EGFR signaling pathway itself can reciprocally regulate global epigenetic modification patterns, affecting changes in the tumor cell phenotype.

### EGFR pathway-related metabolic changes

Activation of the EGFR pathway significantly impacts cellular metabolism, fostering conditions that support rapid tumor growth and potentially contribute to treatment resistance. Metabolomic studies have revealed a series of metabolic changes induced by EGFR pathway activation, providing important clues for understanding the biology of EGFR-driven tumors and developing new therapeutic strategies.

EGFR pathway activation significantly enhances glycolysis, leading to the Warburg effect. This results in increased glucose uptake and significant lactate production. The EGFR pathway achieves this effect by upregulating glucose transporters and activating key glycolytic enzymes [43]. Glutamine metabolism also changes, with EGFR signaling increasing the uptake and utilization of glutamine to support the Tricarboxylic Acid Cycle (TCA) cycle and biosynthesis. Changes in lipid metabolism are another key feature, with EGFR signaling promoting *de novo* fatty acid synthesis and membrane phospholipid synthesis [44].

Amino acid metabolism, particularly that of serine and branched-chain amino acids, is also affected. Changes in nucleotide metabolism support rapid cell proliferation. Alterations in redox balance enhance the antioxidant capacity of a cell. During EGFR-TKI treatment, tumor cell metabolic patterns undergo dynamic changes [45]. Changes in the levels of some metabolites may serve as biomarkers for predicting treatment response. EGFR pathway-related metabolic changes may also affect the tumor microenvironment.

Overall, EGFR pathway-related metabolic changes reflect comprehensive metabolic reprogramming of tumor cells. A deeper understanding of these changes not only helps elucidate the biological characteristics of EGFR-driven tumors but also provides important clues for developing new diagnostic and therapeutic strategies. With advances in metabolomics technology and the integration of multiomics data, we hope to more comprehensively understand the complex interactions between EGFR signaling and cellular metabolism, opening new avenues for precision treatment of NSCLC.

## Resistance and Overcoming Resistance Multimodal Analysis of Resistance Mechanisms

With advances in high-throughput sequencing and bioinformatics, research into the mechanisms underlying resistance to EGFR-TKIs has evolved from single-gene analyses to comprehensive multimodal approaches. This multidimensional analysis reveals complex resistance networks and offers valuable insights for overcoming resistance and developing new therapeutic strategies. The following sections detail the multimodal analysis of EGFR-TKI resistance mechanisms across various omics layers, including genomics, transcriptomics, proteomics, epigenomics, metabolomics, and single-cell technologies.

## Clinical Applications of Multimodal Omics Data in NSCLC Treatment

The integration of multimodal omics data significantly impacts clinical decision-making in NSCLC treatment by enabling physicians to tailor therapies to individual patient profiles. Understanding specific genetic mutations and activated pathways within a patient’s tumor allows the selection of the most effective EGFR-TKIs or the consideration of combination therapies to prevent or address resistance [46]. For instance, patients with exon 19 deletions may respond more favorably to osimertinib, whereas those with exon 20 insertions, often resistant to first- and second-generation TKIs, may benefit from targeted agents like poziotinib or amivantamab, a bispecific antibody targeting both EGFR and MET, which has shown efficacy in patients with EGFR Exon 20 insertion mutations [47,48].

Transcriptomic analyses reveal upregulation of survival pathways, allowing oncologists to incorporate pathway inhibitors, such as PI3K or mTOR inhibitors, alongside EGFR-TKIs [49]. Proteomic analysis uncovers activation of bypass pathways, such as MET amplification or HER2 activation, which may prompt the addition of MET or HER2 inhibitors to EGFR-targeted treatment regimens [50]. Epigenomic studies, by identifying DNA methylation changes, suggest potential benefits of combining epigenetic modifiers with EGFR-TKIs to restore sensitivity in resistant tumors [51]. Metabolomic profiling offers predictive markers, like elevated lactate levels, which can guide metabolic interventions alongside targeted therapy [52].

Beyond therapeutic selection, multimodal omics data also enhances monitoring of treatment efficacy and early detection of resistance. Circulating tumor DNA (ctDNA) assays enable dynamic tracking of mutational changes, allowing timely adjustment of therapeutic strategies when resistance mutations, such as T790M or C797S, emerge [53,54]. These omics-driven insights foster a more personalized, adaptive approach to NSCLC treatment, optimizing outcomes and prolonging patient survival.

## Multimodal Omics Analysis of Resistance Mechanisms

Overcoming resistance to EGFR-TKIs remains a significant challenge in NSCLC treatment. As our understanding of resistance mechanisms deepens, various strategies have been proposed and validated in clinical settings. These include the development of new-generation TKIs, combination targeted therapies, integration of immunotherapy, and other emerging approaches.

Genomics: Beyond the well-known T790M mutation, new resistance mutations are continuously being discovered. Whole-genome and whole-exome sequencing technologies have enabled a comprehensive understanding of genomic changes associated with resistance. For example, the T790M mutation increases ATP affinity, rendering first- and second-generation EGFR-TKIs (such as gefitinib and erlotinib) less effective [55]. The C797S mutation prevents covalent binding of osimertinib to EGFR, resulting in third-generation TKI resistance [56].

Additionally, whole-genome sequencing has revealed complex genomic rearrangements and copy number variations (CNVs), such as EGFR amplification and MET amplification, which are associated with TKI resistance [57]. Notably, the development of liquid biopsy technology allows for the dynamic monitoring of these genomic changes through ctDNA, enabling early detection of resistance and timely intervention.

Transcriptomics: Transcriptomic analysis, primarily through RNA sequencing (RNA-seq), has revealed the activation of multiple signaling pathways in resistant cells. For example, the upregulation of genes associated with epithelial‒mesenchymal transition (EMT) has been confirmed in multiple studies to be related to EGFR-TKI resistance [57]. RNA-seq can also detect the formation of fusion genes, such as the EML4-ALK fusion reported in some cases of EGFR-TKI resistance [58]. Additionally, changes in the expression of long noncoding RNAs (lncRNAs) and microRNAs (miRNAs) have been found to participate in the resistance process. For example, the upregulation of the lncRNA UCA1 may contribute to EGFR-TKI resistance by activating the AKT/mTOR signaling pathway [59]. These transcriptomic changes provide not only new biomarkers of resistance but also a theoretical basis for combined targeted therapy. Table 2 summarizes the EGFR-associated gene expression changes, including upregulated and downregulated genes, feedback regulators, and resistance-related factors that influence the functional impact of EGFR signaling.

**Table 2:** EGFR-associated gene expression patterns

Type of gene expression change	Related genes	Functional impact
Upregulated expression	CCND1, CDK4, BCL2, BIRC5, VEGFA, ANGPT2	Promotes cell proliferation, anti-apoptosis, and angiogenesis
Downregulated expression	BAX, CASP3, CDH1, ITGB1	Inhibits apoptosis and cell adhesion
Feedback regulation	DUSP6, SPRY2	Negative feedback regulation of the EGFR signaling pathway
Resistance-related	MET, HER2, AXL	Bypass activation, may lead to resistance to EGFR-TKIs
Changes in transcription factor activity	c-MYC, E2F1 (increased activity); p53 (activity may be inhibited)	Affects downstream gene expression and cell cycle regulation
Noncoding RNA	Specific microRNAs and long noncoding RNAs	Regulates EGFR expression and downstream gene expression

Proteomics: Proteomics, particularly phosphoproteomics, provides key insights into the remodeling of signaling networks in resistant cells. Proteomics: Proteomics, particularly phosphoproteomics, provides key insights into the remodeling of signaling networks in resistant cells. Through mass spectrometry, researchers have discovered various bypass activation mechanisms, such as the activation of the receptor tyrosine kinases MET, AXL, and IGF1R [60]. These findings directly guide the design of combined targeted therapy strategies [60]. These findings directly guide the design of combined targeted therapy strategies. For example, on the basis of the discovery of MET activation, a treatment strategy combining an EGFR-TKI with a MET inhibitor is being evaluated in clinical trials [61]. Additionally, proteomics has revealed several unexpected mechanisms of resistance, such as changes in the expression of certain cell adhesion molecules that may affect drug sensitivity [62]. Table 3 summarizes the key proteins associated with the EGFR signaling pathway, their functions, and their significance in NSCLC. This table provides a clear overview of the complex molecular interactions that contribute to EGFR activation and resistance.

**Table 3:** EGFR-associated gene expression patterns

Category	Key proteins	Main function/Interaction	Significance in NSCLC
Ligands	EGF, TGF-α, HB-EGF	- Binds to the extracellular domain of EGFR	- Overexpression leads to persistent activation
		- Activates the EGFR signaling pathway	- Autocrine loops promote tumor growth
Receptors	EGFR, HER2, HER3, HER4	- Forms homodimers or heterodimers	- Mutations or amplifications lead to abnormal activation
		- Activates downstream signaling pathways	- Influences treatment response and prognosis
Adaptor proteins	GRB2, SHC, GAB1	- Links receptors to downstream effector molecules	- Regulates signal strength and duration
		- Promotes signal complex formation	- Affects cell proliferation and survival
Downstream effectors	RAS, RAF, MEK, ERK, PI3K, AKT, mTOR, STAT3	- Activates multiple signaling cascades	- Promotes cell proliferation, survival, and migration
		- Regulates gene transcription and protein synthesis	- Potential therapeutic targets
Negative regulators	PTEN, DUSP6, SPRY2, CBL	- Inhibits signal transduction	- Loss of function can lead to overactivation of signaling
		- Promotes receptor internalization and degradation	- Influences treatment resistance
Cross-activation proteins	MET, IGF1R, FGFR, AXL	- Interacts with the EGFR signaling pathway	- Involved in EGFR-TKI resistance mechanisms
		- Activates overlapping downstream pathways	- Provides basis for combined targeted therapy
Nuclear effectors	EGFR, STAT3, β-catenin	- Translocates to the nucleus	- Influences tumor progression and metastasis
		- Directly regulates gene transcription	- May be associated with drug tolerance

Epigenomic: Epigenomics studies have shown that changes in DNA methylation and histone modifications play important roles in EGFR-TKI resistance. Whole-genome methylation sequencing revealed that the methylation status of the promoters of certain resistance-related genes changes, leading to changes in gene expression. For example, high methylation of the PTEN promoter leading to its downregulation may impact EGFR-TKI resistance [63]. Histone modifications, such as acetylation and methylation, have also been found to be involved in the regulation of resistance gene expression [64]. These findings provide a theoretical basis for the application of epigenetic regulators in overcoming EGFR-TKI resistance.

Metabolomic: Metabolomic analysis highlights the significance of metabolic reprogramming in resistant cells. For example, multiple studies have shown that glycolysis is significantly increased in EGFR-TKI-resistant cells, which may be related to the development of resistance [65]. Through techniques such as gas chromatography‒mass spectrometry (GC‒MS) and liquid chromatography‒mass spectrometry (LC‒MS), researchers have also discovered changes in several metabolic pathways, including lipid metabolism and amino acid metabolism [66]. These findings provide not only new ideas for resistance mechanisms but also directions for the development of metabolic targeted therapy strategies. For example, drugs that target glycolysis, such as 2-deoxyglucose (2-DG), have shown potential in preclinical studies to overcome EGFR-TKI resistance [67].

Single-Cell Technologies: The application of single-cell technologies provides unprecedented resolution in understanding the role of tumor heterogeneity in resistance evolution. Single-cell RNA sequencing (scRNA-seq) and single-cell DNA sequencing (scDNA-seq) have revealed the dynamic changes in different subclones during the resistance process. For example, research has shown that under the pressure of EGFR-TKI treatment, a small number of resistant subclones may rapidly expand, eventually leading to clinical resistance [68]. This understanding of subclone dynamics provides important evidence for designing more effective sequential or combined therapy strategies. Additionally, the development of single-cell multiomics technologies, such as Cellular Indexing of Transcriptomes and Epitopes by Sequencing(CITE-seq), allows us to obtain genomic, transcriptomic, and surface protein information at the single-cell level, providing a powerful tool for comprehensively understanding resistance mechanisms.

## Multimodal Omics Analysis of EGFR Pathway in NSCLC and Therapeutic Strategies

Understanding the complexity of EGFR-TKI resistance. By using machine learning and artificial intelligence algorithms, researchers can extract meaningful patterns and associations from massive amounts of multiomics data. For example, research integrating genomic, transcriptomic, and proteomic data has led to the construction of predictive models for EGFR-TKI resistance, which are important for making personalized treatment decisions [69]. Additionally, the integration of multiomics data has aided in the discovery of new potential targets and biomarkers. Table 4 summarizes the main findings and significance of multimodal analyses across genomics, transcriptomics, proteomics, epigenomics, and metabolomics, highlighting their roles in uncovering resistance mechanisms and therapeutic targets.

**Table 4:** Multimodal analysis of resistance mechanisms

Omics level	Main findings	Significance
Genomics	- T790M mutation	Identification of new resistance mutations and genomic changes
	- C797S mutation	
	- EGFR/MET amplification	
	- Genomic rearrangements	
Transcriptomics	- Upregulation of EMT-related genes	Revealing transcriptional regulatory mechanisms related to resistance
	- Fusion genes (e.g., EML4-ALK)	
	- Changes in lncRNA and miRNA expression	
Proteomics	- Activation of bypass signaling pathways	Identification of new therapeutic targets and biomarkers
	- Changes in posttranslational modifications	
Epigenomics	- Changes in DNA methylation patterns	Unveiling epigenetic mechanisms of resistance
	- Histone modification changes	
Metabolomics	- Enhanced glycolysis	Identification of metabolic targets and resistance biomarkers
	- Changes in glutamine metabolism	
	- Reprogramming of lipid metabolism	

However, multiomics analysis also faces several challenges. First, the complexity of data integration is an active research area because of the different characteristics and scales of data from various omics levels. Second, the difficulty and cost of sample acquisition are practical issues, especially for longitudinal studies. Furthermore, how to translate these complex multiomics findings into clinically actionable strategies requires further exploration and validation.

### Strategies to overcome resistance

**Figure 2:**
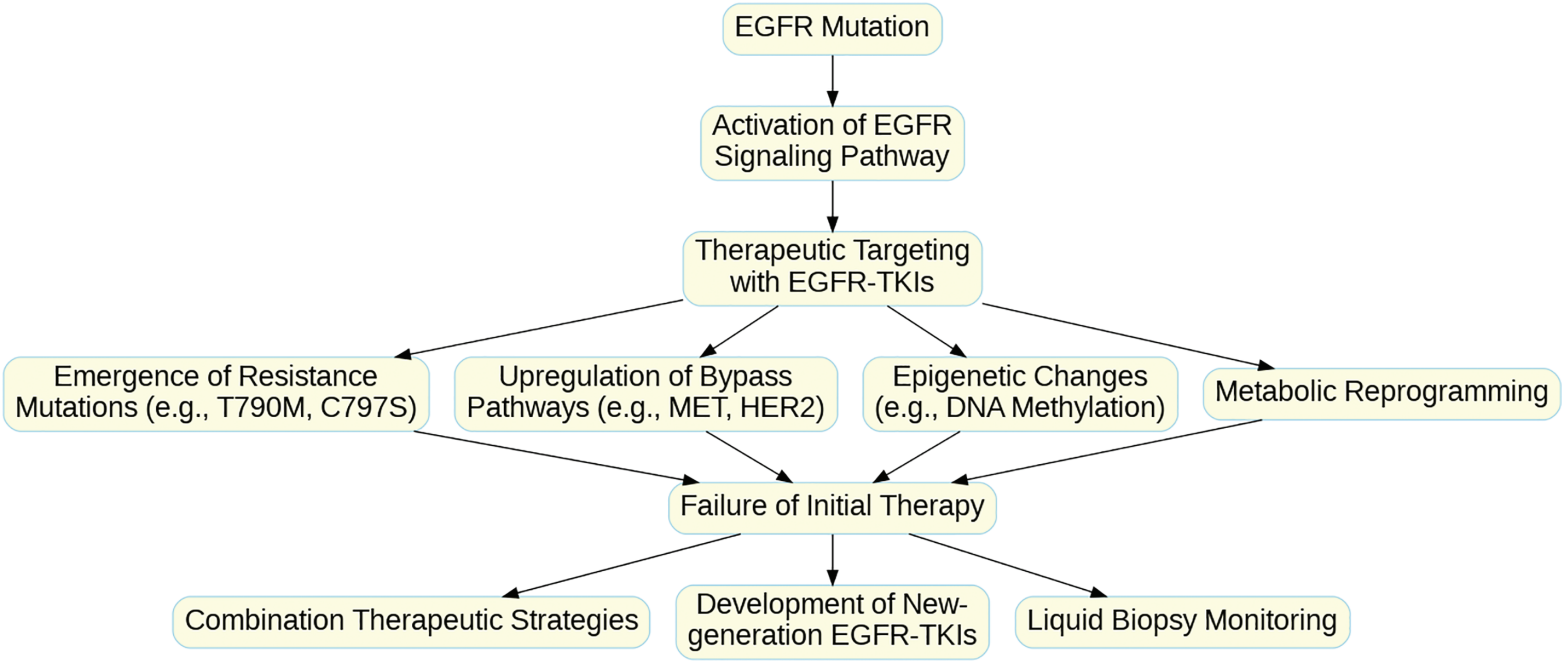
Mechanisms of Resistance to EGFR-TKI Therapy and Strategies for Overcoming Resistance in NSCLC. The flowchart illustrates the progression from initial EGFR mutation and activation of the EGFR signaling pathway to therapeutic targeting with EGFR-TKIs. However, various resistance mechanisms may lead to the failure of initial therapy. These resistance mechanisms include (1) the emergence of secondary resistance mutations (e.g., T790M, C797S), (2) upregulation of bypass signaling pathways (e.g., MET, HER2), (3) epigenetic changes (e.g., DNA methylation), and (4) metabolic reprogramming. These resistance mechanisms collectively contribute to therapy failure, necessitating alternative therapeutic strategies. Post-failure interventions include combination therapies, the development of new-generation EGFR-TKIs, and the use of liquid biopsy monitoring for real-time detection of resistance mutations and treatment adaptation. This multimodal approach aims to enhance treatment efficacy and prolong patient survival in NSCLC.

Overcoming resistance to EGFR-TKIs is a significant challenge in the treatment of non-small cell lung cancer (NSCLC). With a deeper understanding of resistance mechanisms, various strategies have been proposed and validated in clinical settings to counteract resistance. These strategies include the development of new generation TKIs, combination targeted therapies, the integration of immunotherapy, and other emerging approaches. Below, these strategies and their prospects and challenges in clinical applications are discussed in detail. Fig. 2 illustrates the mechanisms of resistance to EGFR-TKI therapy, including secondary mutations, bypass pathway activation, epigenetic changes, and metabolic reprogramming, as well as strategies to overcome resistance, such as combination therapies and liquid biopsy monitoring. And Table 5 summarizes key resistance mechanisms, corresponding overcoming strategies, specific drugs, and their potential advantages, providing a concise overview of targeted interventions for EGFR-TKI resistance.

**Table 5:** Strategies to overcome resistance

Resistance mechanism	Overcoming strategy	Specific methods/Drugs	Potential advantages
T790M mutation	Third-generation EGFR-TKIs	- Osimertinib	- High selectivity for T790M
		- Lazertinib	- Reduced inhibition of wild-type EGFR
Bypass signaling activation (e.g., MET amplification)	Combination targeted therapy	- EGFR-TKI + MET Inhibitor	- Simultaneous inhibition of multiple signaling pathways
		- EGFR-TKI + MEK Inhibitor	- Prevention of compensatory activation
Epigenetic changes	Epigenetic regulators	- HDAC inhibitors	- Reversal of resistance-related gene expression
		- DNA methylation inhibitors	- Enhanced drug sensitivity

The development of new generation TKIs is the most direct strategy to overcome resistance. The success of the third-generation EGFR-TKI osimertinib, which effectively overcomes T790M-mediated resistance, is a prime example. However, facing new resistance mutations such as C797S, fourth-generation EGFR-TKIs are under development. For example, EAI045, an alkylating agent, can covalently bind to the C797 site of EGFR and has shown potential in preclinical studies to overcome both the T790M mutation and the C797S mutation [70]. Additionally, dual or multitarget TKIs, such as poziotinib, which targets EGFR/HER2, and JNJ-61186372, which targets EGFR/MET, are in development [71]. The development of these novel TKIs aims not only to overcome known resistance mechanisms but also to delay the onset of resistance through multitarget inhibition.

Combination targeted therapy is another highly regarded strategy. On the basis of the understanding of bypass activation mechanisms, various combination regimens are being evaluated in clinical trials. For example, the combination of an EGFR-TKI with a MET inhibitor (such as capmatinib) has shown the expected effects on MET amplification-mediated resistance [62]. The TATTON study evaluated the effects of osimertinib combined with savolitinib and reported significant antitumor activity in patients with dual-positive EGFR/MET [72]. Another example is the combination of an EGFR-TKI with an antiangiogenic agent, such as erlotinib combined with bevacizumab, which showed superior PFS benefits in the NEJ026 study compared with monotherapy [73]. These combination strategies aim not only to counter existing resistance mechanisms but also to delay the emergence of resistance by simultaneously inhibiting multiple critical pathways.

The integration of immunotherapy has been a major breakthrough in the field of lung cancer treatment in recent years, but its application in EGFR-mutant NSCLC still faces challenges. Although monotherapy with PD-1/PD-L1 inhibitors has limited efficacy in these patients, the combination of immunotherapy with EGFR-TKIs is being actively explored. For example, the IMpower150 study revealed that the triplet regimen of atezolizumab combined with bevacizumab and chemotherapy might improve survival in patients with EGFR mutations [74]. Moreover, innovative immunotherapeutic strategies, such as bispecific antibodies and CAR-T-cell therapies, are also being evaluated in EGFR-mutant NSCLC. For example, a bispecific antibody targeting EGFR and CD3, AMG 757, showed promising activity in early clinical trials [75].

However, overcoming EGFR-TKI resistance still faces many challenges. First, the complexity of tumor heterogeneity and evolution means that a single strategy is unlikely to completely resolve resistance issues. Second, multitarget inhibition or combination therapy may increase toxicity, and balancing efficacy with safety is a key issue. Additionally, some resistance mechanisms, such as small cell lung cancer transformation, still lack effective targeted strategies.

## Improvements in Multimodal Data Analysis Methods

With the rapid development of high-throughput sequencing technologies, the generation of multimodal data has far exceeded our ability to analyze and understand these data. Therefore, improving multimodal data analysis methods will be a key direction for future research. First, we need to develop more efficient data integration algorithms to fully utilize information from genomics, transcriptomics, proteomics, epigenomics, and other levels. For example, graph-theoretical approaches could provide new insights for integrating different types of omics data, allowing the construction of more comprehensive molecular network models.

Second, the application of machine learning and deep learning techniques in multimodal data analysis will further expand. For example, convolutional neural networks (CNNs) and recurrent neural networks (RNNs) can be used to extract spatiotemporal patterns from complex multimodal data, helping us understand the dynamic processes of EGFR-TKI resistance. Additionally, transfer learning techniques may allow us to apply models trained on large-scale public datasets to smaller clinical datasets, thus improving prediction accuracy. Another important direction is the development of more powerful single-cell multimodal analysis methods. With advancements in single-cell sequencing technologies, we can obtain multidimensional information such as genomics, transcriptomics, and epigenomics at the single-cell level. However, effectively integrating these high-dimensional, high-noise data remains a challenge. The development of analysis tools specifically for single-cell multimodal data, such as improved dimensionality reduction algorithms and trajectory inference methods, will help us better understand tumor heterogeneity and resistance evolution.

Finally, visualization and interpretation of multimodal data also need innovation. The development of intuitive, interactive visualization tools can help researchers and clinicians better understand and utilize complex multimodal data. For example, multidimensional data visualization systems based on virtual reality (VR) or augmented reality (AR) technologies might provide new perspectives for exploring complex biological networks.

### Applications of liquid biopsy in the detection of EGFR mutations

Liquid biopsy technologies, particularly the detection of circulating tumor DNA (ctDNA), are transforming the detection and monitoring of EGFR mutations. In the future, we expect significant improvements in the sensitivity, specificity, and range of this technology. For example, further optimization of digital PCR technology might allow us to detect lower abundances of EGFR mutations, enabling ultraearly diagnosis and monitoring of minimal residual disease (MRD) [76]. Additionally, improvements in ctDNA sequencing technology might allow the detection of multiple resistance-related mutations in a single liquid biopsy, providing more comprehensive resistance mechanism information.

Another important direction is the expansion of liquid biopsy sample types. In addition to blood, other bodily fluids, such as urine, cerebrospinal fluid, and pleural effusion, may also be valuable sources of liquid biopsy. For example, cerebrospinal fluid ctDNA analysis might provide more accurate resistance information than blood ctDNA analysis for patients with central nervous system metastases after EGFR-TKI treatment [77]. The development of optimized protocols for these different sample types will be a focus of future research.

The clinical application scenarios for liquid biopsy will also further expand. In addition to initial diagnosis and resistance detection, liquid biopsy could play a significant role in monitoring treatment response, prognosis assessment, and recurrence prediction. For example, by dynamically monitoring ctDNA levels and mutation spectra, we might be able to predict the efficacy of EGFR-TKI treatment early and adjust treatment strategies in a timely manner [78]. Additionally, integrating liquid biopsy with imaging and other clinical indicators to develop comprehensive disease monitoring systems will be key to improving patient management.

Finally, standardization of liquid biopsy data and large-scale data sharing are crucial. Establishing uniform standards for liquid biopsy data collection, analysis, and reporting will facilitate comparisons and meta-analyses between different studies. Moreover, creating a large-scale liquid biopsy database and linking it with clinical information and treatment outcomes will provide valuable resources for developing more accurate predictive models.

### Potential applications of artificial intelligence in EGFR-targeted therapy

Artificial intelligence (AI), especially machine learning and deep learning technologies, has shown tremendous potential in various aspects of EGFR-targeted therapy. First, in the field of drug development, AI can accelerate the design and screening processes for new EGFR-TKIs. For example, deep learning-based molecular generation models might be able to design novel inhibitors targeted against specific resistance mutations [79]. Additionally, AI-driven virtual screening technologies might help us identify compounds with anti-EGFR activity from existing drug libraries, providing new opportunities for drug repositioning.

In terms of clinical decision support, AI systems could transform the practice of personalized treatment. By integrating multidimensional information such as a patient’s genomic data, clinical features, imaging data, and treatment history, AI models might be able to predict individual patients’ responses to different EGFR-TKIs and their risks of resistance, thus guiding the selection of optimal treatment strategies. For example, deep learning-based radiomic models might be able to extract features related to the EGFR mutation status and TKI sensitivity from Computed Tomography (CT) or Positron Emission Tomography-Computed Tomography (PET-CT) images, assisting in diagnosis and treatment decisions [80]. AI could also offer valuable insights for optimizing drug combinations and sequential treatment strategies. By simulating the effects of different drug combinations and administration sequences on tumor evolution, AI models might help design treatment strategies that can maximally delay resistance. Additionally, reinforcement learning algorithms might be used to develop adaptable dosing strategies, dynamically adjusting treatment plans on the basis of real-time patient responses.

Finally, the application of AI in the analysis of real-world data is also promising. By analyzing large-scale electronic health records and insurance data, AI might help us better understand the usage patterns, efficacy, and safety of EGFR-TKIs in the real world, providing important references for the formulation of clinical guidelines and health policies.

### International perspectives on EGFR-targeted therapy

EGFR-targeted therapy has made significant progress in the treatment of NSCLC, but its application and effectiveness vary significantly worldwide. These differences are manifested mainly in EGFR mutation detection, treatment strategy selection, efficacy assessment standards, and the adoption of new technologies.

EGFR mutation detection methods and prevalence vary by region. Western countries generally use next-generation sequencing (NGS) technology, Asian countries often use Polymerase Chain Reaction (PCR) methods, and developing countries rely mainly on immunohistochemistry and real-time PCR. An international survey revealed that EGFR testing rates vary from less than 10% to nearly 100%, with higher rates in high-income countries. This disparity directly affects patients’ access to precision treatment.

There are also regional differences in treatment strategy selection. The US FDA has approved osimertinib as a first-line treatment; most European countries follow similar guidelines, and Asian countries use multiple generations of EGFR-TKIs. Recent international multicenter studies, such as FLAURA2 and MARIPOSA, have evaluated new combination treatment strategies, but participation and treatment models vary by region.

There are significant differences in medical insurance coverage and the speed at which new technologies are adopted. Private insurance and government medical programs in the US provide comprehensive coverage for EGFR-TKIs, which are provided through national health services in Europe, and several Asian countries have included multiple EGFR-TKIs in medical insurance. However, the proliferation of new technologies, such as liquid biopsy and NGS, still faces challenges in developing countries. Innovative treatment clinical trials are concentrated in North America, Western Europe, and East Asia, limiting participation opportunities for patients in other regions.

In summary, the global disparities in EGFR-targeted therapy reflect differences in economic development, health policy, and technological infrastructure. Strengthening international cooperation and resource sharing while developing treatment guidelines suitable for local regions will be key to improving the treatment level of NSCLC patients worldwide. Only through concerted efforts can advancements in EGFR-targeted therapy benefit more patients and ultimately improve their quality of life and prognosis.

## Conclusion

In conclusion, the integration of multidisciplinary and multitechnological advances heralds a transformative era for EGFR-targeted therapies in NSCLC. However, significant obstacles such as the high costs, complexity, and data integration challenges must be overcome to realize the clinical potential of omics analyses. Future innovations are anticipated to leverage artificial intelligence and liquid biopsy technologies, enhancing the precision and dynamic monitoring of disease states. These developments promise not only to refine our understanding of resistance mechanisms but also to guide the evolution of personalized medicine. To transition these technologies from research to bedside, collaborative efforts in technological development, validation studies, and regulatory frameworks are essential. We are on the cusp of major breakthroughs that will potentially redefine the management of EGFR-mutant NSCLC, driven by ongoing research and technological progress.

## Data Availability

Data sharing is not applicable to this article as no datasets were generated or analyzed during the current study. The review is based on previously published articles, which are cited accordingly in the reference section.

## Abbreviation

EGFR: Epidermal Growth Factor Receptor
TKI: Tyrosine Kinase Inhibitor
PFS: Progression-Free Survival
MET: Mesenchymal-Epithelial Transition
GATK: Genome Analysis Toolkit
ANNOVAR: ANNOtate VARiation
STAR: Spliced Transcripts Alignment to a Reference
LC-MS: Liquid Chromatography-Mass Spectrometry
GC-MS: Gas Chromatography-Mass Spectrometry
ctDNA: Circulating Tumor DNA
HER2: Human Epidermal Growth Factor Receptor 2
AXL: AXL Receptor Tyrosine Kinase
CNVs: Copy Number Variations
EMT: Epithelial‒Mesenchymal Transition
RNA-seq: RNA Sequencing
lncRNAs: Long Noncoding RNAs
miRNAs: MicroRNAs
2-DG: 2-deoxyglucose
scRNA-seq: Single-cell RNA Sequencing
scDNA-seq: Single-cell DNA Sequencing
CITE-seq: Cellular Indexing of Transcriptomes and Epitopes by Sequencing
MRD: Minimal Residual Disease
CT: Computed Tomography
PET-CT: Positron Emission Tomography-Computed Tomography
NGS: Next-Generation Sequencing
PCR: Polymerase Chain Reaction
